# Unusual clinicopathological presentation of nontraumatic cerebral fat embolism: Two-case report

**DOI:** 10.1097/MD.0000000000019574

**Published:** 2020-03-20

**Authors:** Hye Seung Lee, Jeong-Jin Park, Hong Gee Roh, So Dug Lim

**Affiliations:** a Department of Pathology, Konkuk University School of Medicine, Seoul, Republic of Korea; b Department of Pathology, Korea Clinical Laboratory, Seoul, Republic of Korea; c Department of Neurology; d Department of Radiology, Konkuk University School of Medicine, Seoul, Republic of Korea.

**Keywords:** basilar artery, cerebral infarction, embolism, intracranial embolism, middle cerebral artery

## Abstract

**Rationale:**

Fat embolism syndrome (FES) is characterized by the classical triad of cerebral, respiratory, and cutaneous manifestations. In contrast, cerebral fat embolism (CFE), corresponding to incomplete pure type FES, is much rarer and usually follows trauma. CFE typically shows a “starfield” pattern on diffusion-weighted magnetic resonance imaging due to the involvement of multiple small arteries. We report 2 unusual cases of CFE that showed a nontraumatic etiology and the involvement of a single dominant cerebral artery.

**Patient concerns:**

Case 1 was a 33-year-old woman without a history of trauma who visited the emergency room due to hemiparesis and hemisensory deficits. She was a heavy smoker and had used oral contraceptives for several years. Most importantly, she had 2 experiences of autologous fat grafting 2 months previously. Magnetic resonance angiography (MRA) revealed acute occlusion of the right middle cerebral artery. Case 2 was an 80-year-old man suddenly presented with dizziness, ataxia, and left-sided sensorimotor dysfunction. He had a history of hypertension, untreated atrial fibrillation, and chronic alcoholism. MRA demonstrated the occlusion of the distal basilar artery.

**Diagnosis:**

Case 1: Microscopic findings demonstrated variable sized fat vacuoles intermixed with moderate amounts of thrombi. Case 2: Histologically, mature adipocytes were intermingled with fibrin, blood cells, and a fragment of entrapped soft tissue resembling the vessel wall.

**Intervention:**

Case 1 and 2 underwent aspirational thrombectomy guided by transfemoral cerebral angiography.

**Outcome:**

Case 1 recovered well but Case 2 still suffers from gait ataxia.

**Lessons:**

CFE can rarely occur in various nontraumatic conditions, with or without evident etiology. Furthermore, it may not show characteristic clinicopathological manifestations. Therefore, careful follow up of those who have undergone procedures that are likely to trigger FES or who have hemodynamic or hypercoagulable risk factors is needed.

## 1 Introduction

Fat emboli are traveling lipid particles or droplets in the blood circulation, generally introduced by long bone fracture after major trauma. Fat embolism is a multistep process with a broader meaning not only including fat emboli passing into the circulation but also lodging within a vessel. This may or may not produce clinical symptoms, and the severity varies depending on the size and amount of fat particles.^[1]^ Fat embolism has a prevalence ranging from 0.1% to 11%, and the severity varies depending on the size and amount of fat particles.^[2–4]^

Although fat embolism is usually asymptomatic, fat embolism syndrome (FES) occurs in 10% of cases of fat embolism and can result in serious symptoms including multiorgan dysfunction. FES is classically characterized by the triad of pulmonary compromise, petechial hemorrhage, and neurological manifestations.^[5]^ It most commonly affects the lungs, followed by the brain.^[6]^ Infrequently, neurological symptoms may predominate without other manifestations, in which case it is referred to as “cerebral fat embolism” (CFE). Because there are no signs or symptoms of other organs, CFE can be easily misdiagnosed. Neuroimaging abnormalities on diffusion-weighted magnetic resonance imaging (DW-MRI) could help the diagnosis of CFE, which is characterized by a typical “starfield pattern,” indicating bilateral multifocal microinfarction of the small cerebral vessels.^[7]^

Here, we report 2 very rare cases of pathologically proven nontraumatic CFEs, in the form of single major cerebral artery occlusions and discuss its pathogenesis and clinical significance. The informed consent has been waivered for this manuscript by the institutional review board (IRB) of Konkuk University Medical Center (KUMC 2019-07-005).

## 2 Case reports

### 2.1 Case 1

A 33-year-old woman, with a body mass index of 24.9 kg/m^2^ was admitted to our hospital via the emergency room due to sudden onset of altered consciousness and drooling. At admission, she was relatively alert and her vital signs were stable. On neurologic examination, she showed left facial palsy, left arm and leg drift, dense left-sided sensory loss, mild to moderate dysarthria, and partial neglect, indicating neocortical dysfunction. The patient had no history of trauma, epilepsy, or cerebral hemorrhage but had a history of 2 procedures of gluteal augmentation 2 months previously. She underwent massive liposuction of her arms, legs, and abdomen, and autologous fat was injected into her buttocks. Additionally, she had been taking oral contraceptives over 5 years and was a heavy smoker (15 pack × years).

The whole blood and blood chemistry tests, including lipid profile, revealed results within normal values. The hypercoagulability panel revealed a decreased protein S antigen level (54%, normal: 60–150%), protein S antigen activity (38%, normal 58.7–119.2%), and anti-thrombin level (54%, normal: 80–120%). The total protein C antigen level, serum homocysteine level, antinuclear factor antibody, anti-double stranded deoxyribonucleic acid (anti-dsDNA) antibody, and anti-cardiolipin antibody were within normal limits.

**Figure 1:**
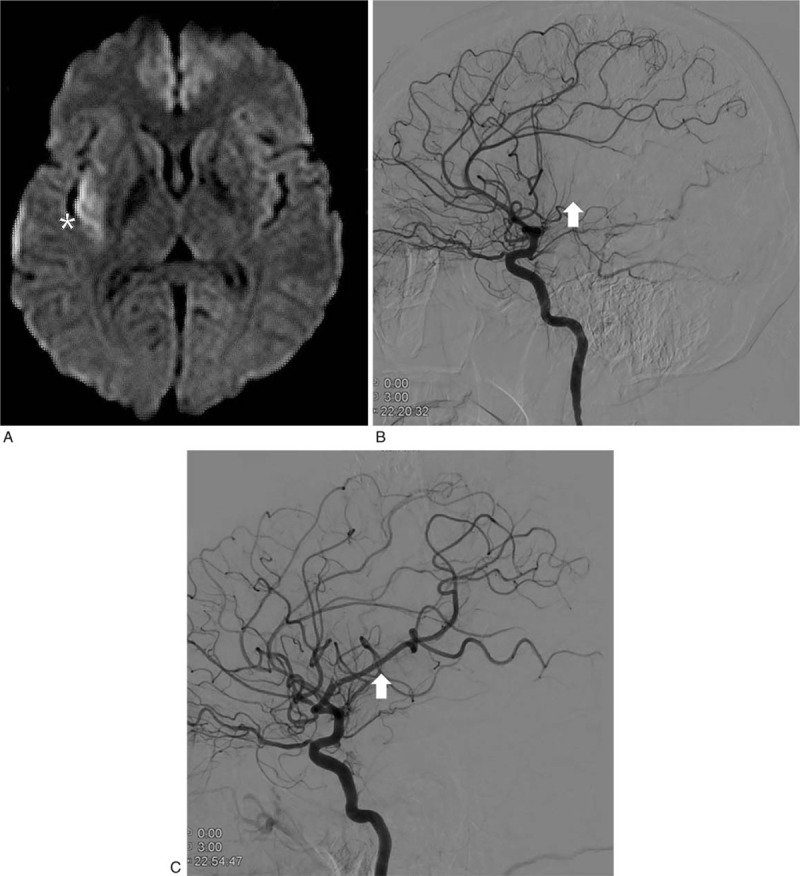
A case of nontraumatic cerebral fat embolism in a young woman (case 1) that presented with right middle cerebral artery occlusion after autologous fat grafting. (A) Diffusion-weighted magnetic resonance imaging reveals localized delay and decrease of perfusion in the right temporoparietal area (asterisk). (B) Trans-femoral cerebral angiography reveals occlusion post-bifurcation of the M1/M2 junction of the right middle cerebral artery (white arrow). (C) Post-interventional angiography highlights successful recanalization (white arrow).

On imaging studies, computed tomography (CT) showed no definite early ischemic change in the brain. However, DW-MRI revealed multifocal slow diffusion to the right temporo-parietal region with focal cerebral blood volume defects in the right insula and angular region (Fig. 1A). The lesion was demonstrated to be an acute occlusion of the right middle cerebral artery (MCA) on magnetic resonance angiography (MRA) and following cerebral angiography using a transfemoral approach (Fig. 1B). The coaxial system was comprised of a Penumbra ACE68 reperfusion catheter (Penumbra, Inc., CA) and Marksman microcatheter (Igiasi S.A., Greece). Forced arterial suction thrombectomy was then performed using a 50-mL syringe on the right MCA occlusion 3 hours after symptom onset. Antiplatelet medication was infused into the artery 3 times, and most MCA branches were finally revisualized (Fig. 1C).

**Figure 2:**
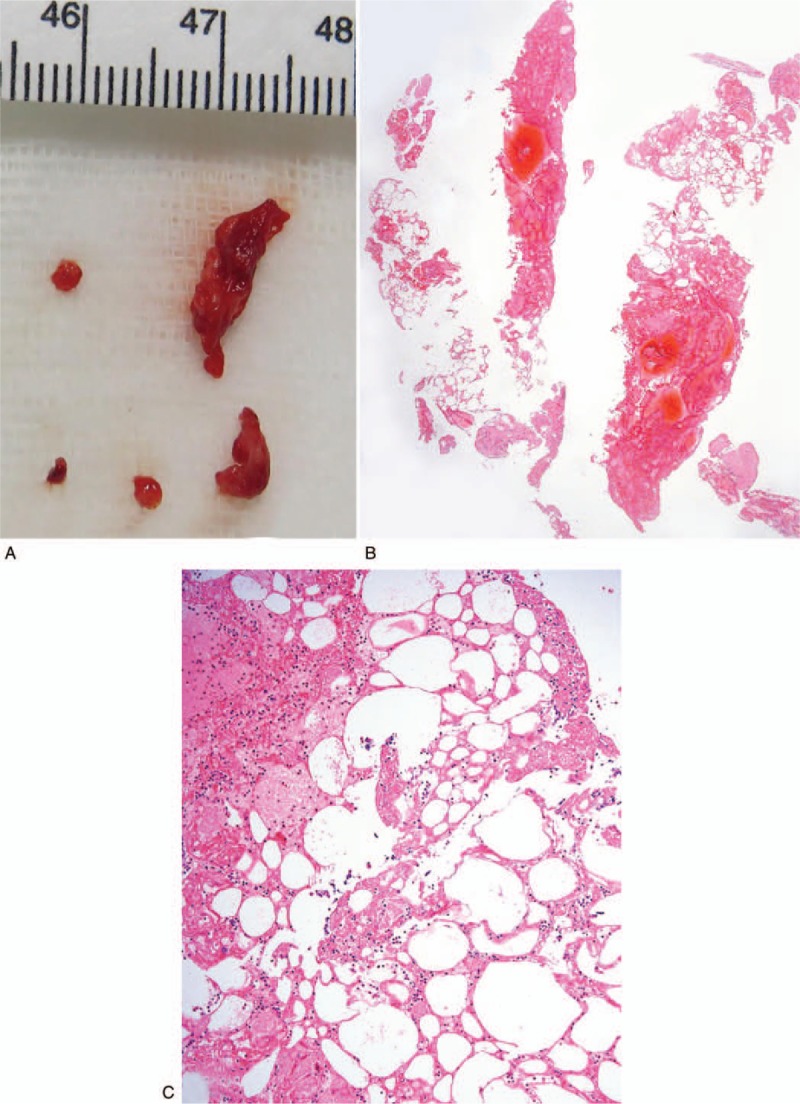
Gross and microscopic findings in a young woman (case 1) after autologous fat grafting. (A) The aspirated material from the right middle cerebral artery shows several pieces of soft tissue of yellow to reddish color. (B) On a scan power view, moderate amounts of admixed fibrinoid material and some fat tissue are seen. (C) On medium magnification, mature adipose tissues are intermingled with fibrin and mixed blood cells.

Gross examination revealed fragmented fatty tissues with a yellow to red-brown color (Fig. 2A). Microscopically, these corresponded to variably sized adipocyte-like cells intermixed with newly formed thrombi (Fig. 2B). At high magnification, the vacuoles were demonstrated to be mature adipocytes (Fig. 2C). After neurointervention, several studies including 2-dimensional echocardiography, carotid sonography, transcranial doppler, and coronary CT were performed to find the cause of the embolic stroke. However, no abnormal findings were observed.

The patient's recovery was uneventful after intervention and she was discharged in a stable condition on her fifth day after admission. During a 1-year follow-up period, almost all neurological deficits recovered.

### 2.2 Case 2

An 80-year-old man visited the emergency room due to a 1-hour history of sudden onset dizziness and diplopia. Upon arrival to the emergency department, he had a high blood pressure of 167/90 mmHg. Other vital signs were determined to be stable with a body temperature of 36.7 °C, pulse of 95 beats per minute, respiratory rate of 17 breaths per minute, and oxygen saturation of 95%. The patient was alert, and talking with a Glasgow Coma Scale (GCS) score of 15. He did not present with any history of trauma, but had a medical history of hypertension under treatment, untreated atrial fibrillation due to chronic subdural hematoma caused by a fall 3 years previously, and chronic alcoholism. Neurologic examination revealed left-sided sensorimotor changes including gaze limitation of the medial eye, perioral sensory change, leg weakness, and mild dysarthria. Therefore, ischemic or hemorrhagic stroke of the right medial pontine tegmental area was suspected.

**Figure 3:**
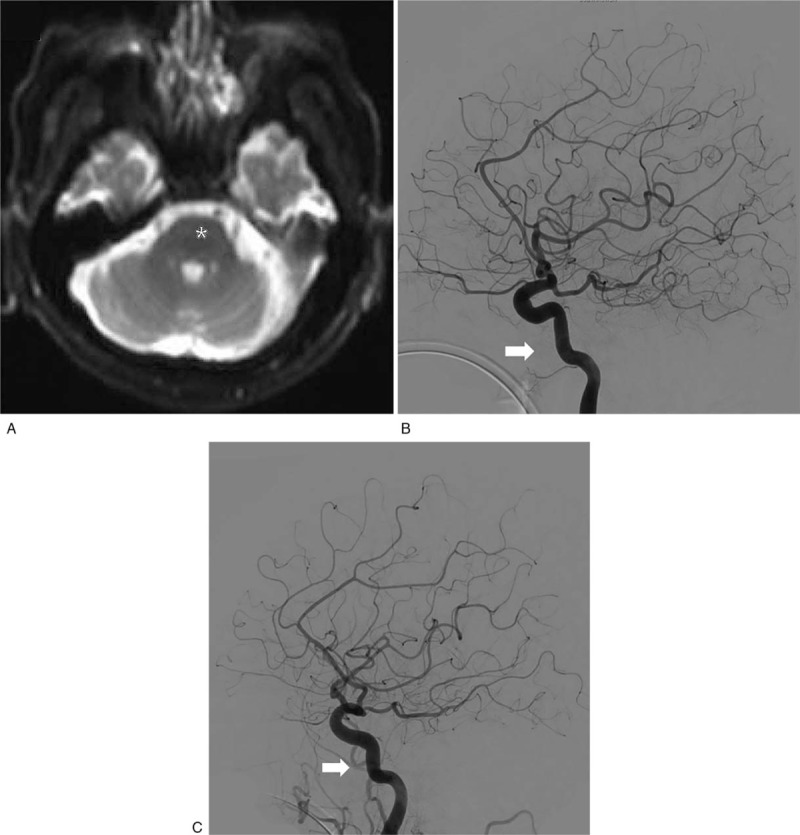
A case of nontraumatic cerebral fat embolism in an old man (case 2) that presented with distal basilar artery infarction. (A) Diffusion-weighted magnetic resonance imaging reveals very subtle hyperintensities in the right ventral pons and right medial longitudinal fasciculus (asterisk). (B) Trans-femoral cerebral angiography reveals short segmental occlusion in the distal basilar artery trunk and V4 segment of the left vertebral artery (white arrow). (C) Post-interventional angiography reveals almost complete recanalization of the occluded areas (white arrow).

The results of the clinical chemistry showed high triglyceride (180 mg/dL, normal: 45–150 mg/dL), and high uric acid levels (8.0 mg/dL, normal: 4–7 mg/dL). The values of the whole blood tests were within normal ranges. The DW-MRI scans documented only subtle hyperintensities in the right ventral pons and right medial longitudinal fasciculus (Fig. 3A). However, subsequent MRA demonstrated retrograde filling of the terminal basilar artery and superior cerebellar arteries (SCAs), indicating occlusion of the distal basilar artery proximal to the SCA origins. Altogether, embolic or thrombotic stroke was suspected.

Using x-ray guidance, transfemoral cerebral angiography (TFCA) and following intra-arterial thrombectomy were performed. The right common femoral artery was punctured under local anesthesia, and a 5 French sheath was inserted. Bilateral internal carotid arteries, left subclavian-vertebral artery, and right vertebral artery angiography were performed using a 5 French Berenstein catheter. Contrast material injection revealed short segmental occlusion in the distal basilar artery trunk, mild stenosis in the right vertebral artery orifice, and left vertebral artery occlusion in the V4 segment after branching the posterior inferior cerebellar artery (Fig. 3B).

Aspiration thrombectomy for the distal basilar artery occlusion was performed. After changing to an 8 French femoral sheath, a 125 cm 5 French Berenstein catheter was inserted into the 8 French guiding catheter. The guiding catheter was placed in the subclavian artery near the right vertebral artery orifice. The coaxial catheter system was made by placing a Marksman microcatheter in a 160 cm Penumbra ACE68 Reperfusion catheter (ACE68). The guiding catheter was advanced into the distal second segment of the vertebral artery and the ACE68 was successfully advanced further into the distal basilar artery. Aspiration with a 50-mL syringe was performed and the basilar artery was recanalized completely (Fig. 3C).

**Figure 4:**
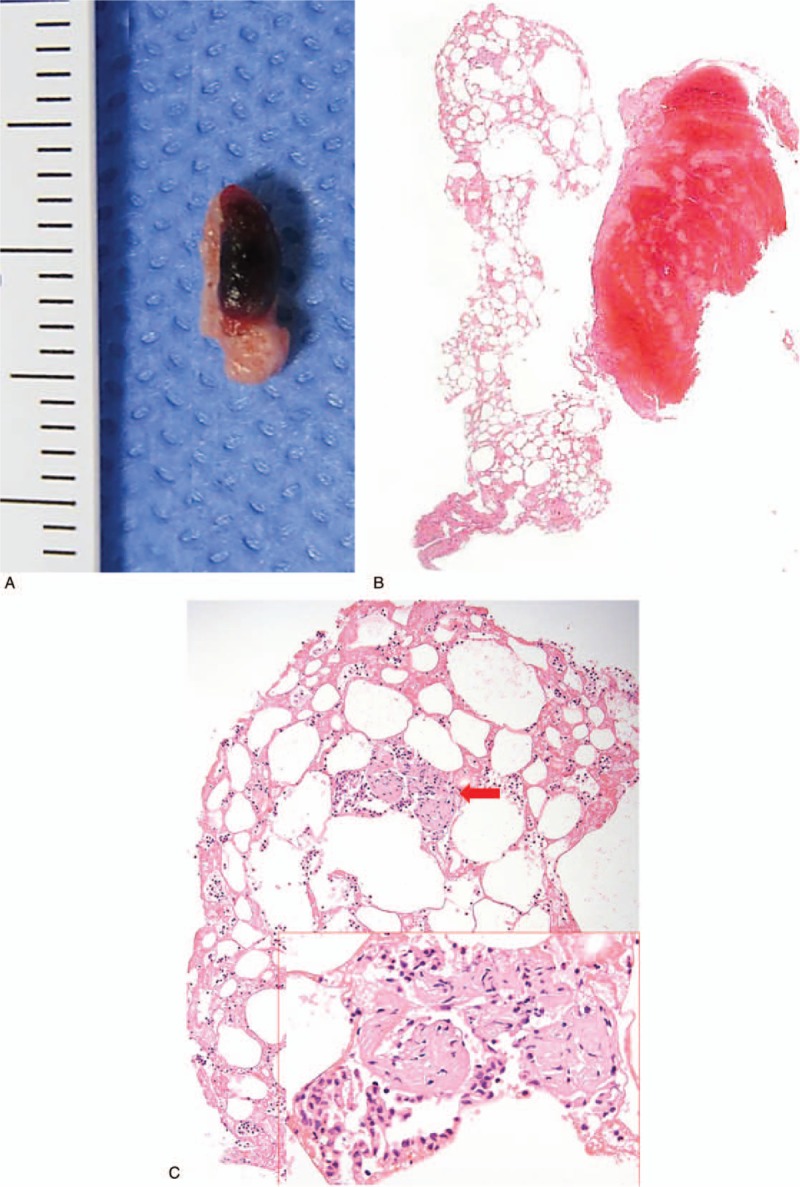
Gross and microscopic findings in an old man (case 2). (A) The aspirated material from the distal basilar artery shows an 8-mm-sized lump of yellowish adipose tissue with an attached blood clot. (B) The specimen shows morphologic correlation on a low magnification image. (C) Variably sized mature adipocytes are intermixed with fibrin and blood cells and surrounding a small fragment of entrapped soft tissue (red arrow). An enlarged image of the fragment reveals a histology resembling vessel wall.

The removed material consisted of a large pale yellow-colored soft tissue with adherent discrete collections of clotted blood (Fig. 4A). Microscopically, those materials were demonstrated to be mature adipose tissue and fibrinoid thrombi on a low-power view (Fig. 4B). The fatty tissues were intermingled with a mixed cell population of blood cells (Fig. 4C). Interestingly, a fragment of the entrapped soft tissue resembling the vascular wall was found, representing the tunica media and tunica intima (Fig. 4C, inset).

The post-interventional magnetic resonance imaging (MRI) showed non-specific findings and he recovered uneventfully until the discharge date. The patient showed a slow neurological recovery during the 1-year follow-up and still has ongoing gait disturbance.

## 3 Discussion

To date, 2 major and 1 minor theory have been postulated as the pathogenesis of FES. They provide different explanations about the route of the fat emboli to the distant organ. The first is a “mechanical theory,” which is more suitable for traumatic causes. The fat particle is released into the venous system from ruptured bone after trauma, and enters the pulmonary circulation.^[8]^ Once a fat vacuole reaches the pulmonary vasculature, it would go through into the systemic circulation via cardio-pulmonary shunts, such as a patent foramen ovale, and go on to distant organs.^[9]^ However, there is also a study of mechanically-induced FES without shunts, which demonstrated bypass of fat globules under high pulmonary artery pressure.^[10]^

The second is the “biochemical theory,” which better explains nontraumatic causes. In a stressed state, catecholamine secretion increases and results in the incorporation of a large amount of fat into the blood. The activated lipase then degrades a triglyceride into 1 glycerol and 3 free fatty acids (FFAs). The large amount of released FFAs may freely cross the blood brain barrier, and enter numerous small to medium sized cerebral vessels.^[11]^

Finally, there is a “coagulation theory.”^[12]^ Tissue thromboplastin and the complement system become activated by trauma, systemic inflammation, or soft tissue injury.^[13]^ The fibrin and its degradation products produced by coagulation cascade then clump with fat globules, platelets, and inflammatory cells, and cause endothelial cell damage. The endothelial cell damage and release of vasoactive substances increase vascular permeability, and stimulate migration of fat vacuoles into the systemic circulation. Once fat emboli are settled on the vessel wall of a distant organ, the mechanism thereafter is similar. Inflammation increases local pressure, resulting in accelerated aggregation of fat emboli, and following further inflammatory reaction may induce sudden vascular occlusion.^[14]^

In this report, patient histories and microscopic findings support the biochemical and coagulation theories. Case 1 and 2 had no history of cardiopulmonary shunts or trauma. Case 1 revealed a mixture of mature adipocytes and fibrin, implying abrupt vascular damage. Case 2 included an entrapped fragment of spindle cells suggestive of the vascular wall, and this would be indirect evidence of endothelial cell damage and associated inflammation. In addition, case 1 and 2 showed a large fat bolus of aspirated material, which supports a biochemical mechanism. The predisposing factors that can trigger FES are mainly divided into traumatic and nontraumatic causes and are described in Table 1.^[6]^ Nontraumatic factors can be divided into 2 categories, medical disease and surgery/procedure. In recent years, the number of cases of nontraumatic FES has increased as the number of performed procedures has increased.^[15]^ The number of liposuction procedures has increased by 124% since it was first reported in 1997.^[16]^ Up to now, the number of reports of liposuction-induced FES has been <20.^[17]^ Nonetheless, the associated overall mortality has been reported as approximately 10% to 15%.^[10]^

**Table 1:**
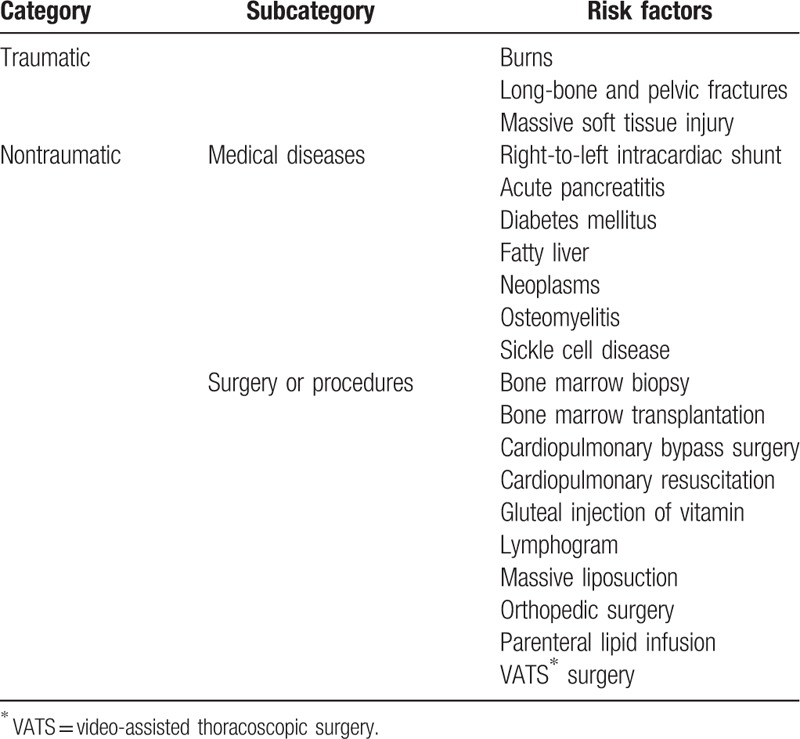
Traumatic and nontraumatic predisposing factors of fat embolism syndrome.

The first case received autologous fat grafts from many parts of the body and this would have increased the risk of CFE. Whether the probability of FES increases with the amount of liposuction is still controversial.^[18]^ She had additional risk factors for FES. Smoking, long-term use of oral contraceptive (>5 years), and her decreased protein S antigen and anti-thrombin levels indicate her hypercoagulable state. Smoking increases serum catecholamine levels and promotes the release of FFAs.^[19]^ All these factors may have contributed to the degradation of fat vacuoles and amplification of the coagulation cascade. A moderate amount of newly formed thrombus adjacent to the fat tissue supports this speculation.^[20,21]^

The second patient had no trauma history and his skull x-ray image revealed no fracture or other significant abnormalites. However, he had some factors that could affect endothelial dysfunction. Ntaios et al^[22]^ found that 30% of patients with embolic stroke of undetermined source (ESUS) had atrial fibrillation. Furthermore, hypertension may provide a hemodynamic force, and make emboli aggregate more easily due to vasoconstriction in distant organs. Hyperlipidemia and chronic alcoholism stimulate the coagulation cascade and induce endothelial damage. Specifically, hypertriglyceridemia facilitates lipid metabolism to breakdown triglycerides into FFAs, and this supports a biochemical mechanism. Although liver ultrasonography or abdominal CT was not tested in this patient, it has been reported that fat metabolism is altered in the fatty liver derived from chronic alcoholism and large amounts of FFAs circulate in the bloodstream as a result.^[23]^

In 1970, Gurd^[24]^ proposed clinical diagnostic criteria for FES, with the 3 major criteria including respiratory distress, neurologic signs, and petechial rash (Table 2). After several years, Gurd and Wilson^[25]^ proposed modified clinical diagnostic criteria for FES that are more specific and more commonly in use at present (Table 2). In 1977, Sevitt^[26]^ classified FES into 3 types, eruption, complete, and incomplete, according to their clinical features. Among them, the incomplete type is subclassified into 3 types; pure cerebral, pure pulmonary, and mixed. Accordingly, CFE belongs to the incomplete and pure cerebral type.

**Table 2:**
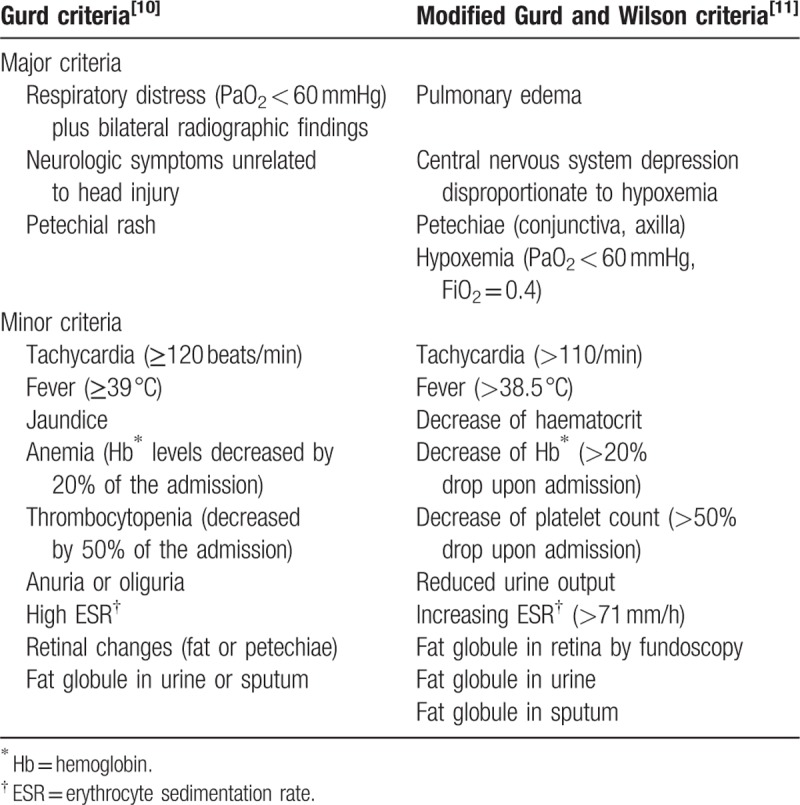
Diagnostic criteria for fat embolism syndrome.

It is easier to diagnose pulmonary and mixed types of FES. However, if only neurologic symptoms are present, it is very difficult to diagnose. Neurologic dysfunction is present in about 80% in the early stages of FES but generally accompany respiratory symptoms. The common neurologic symptoms consist of altered consciousness, dementia, convulsion, ipsilateral numbness, and paralysis.^[11]^ Respiratory distress is present in 75% of patients in the early stage, presenting as dyspnea, tachypnea, and hypoxemia. Cutaneous manifestations, usually nonpalpable petechiae distributed preferentially in the conjunctiva, axillae, and upper chest, are present in 50% of cases.^[27]^ Both incomplete pure type and complete type (<40%) are relatively rare.

More than 80% of acute phase CFE typically shows a characteristic MRI finding, the “starfield pattern,” bilaterally scattered high-intensity multifocal spots on DW-MRI.^[28]^ Kuo et al^[29]^ reviewed 38 magnetic resonance images from 27 CFE patients, and divided them into 3 types in association with time. The most common pattern is type 1 (61.5%), synonymous with the “starfield pattern,” showing scattered cytotoxic edema. This type tends to be reversible, and has better clinical outcome. In order of frequency, the others in the acute stage show petechial hemorrhage of the white matter (type 2C), vasogenic edema (type 2B), and confluent cytotoxic edema (type 2A). Type 3 represents chronic sequelae.

In the present report, the first case showed unilateral multifocal hyperintensities on DW-MRI. The second case only showed subtle hyperintensities in a localized area. Case 1 and 2 showed unexpected imaging findings that did not correspond to type 1–3 categories, which could masquerade as typical thrombotic or thromboembolic stroke. This is very interesting because those patterns and predominant single major artery involvement are rare in typical FES. In the literature, only 1 of 6 cases of CFE revealed left MCA occlusion after facial fat grafting.^[30]^

In summary, our 2 nontraumatic CFE cases share some unusual clinicopathological features: absence of a shunt, considerable time delay from the point of exposure to the risk factors to the time of the clinical emergency, involvement of a single artery, formation of a large fat globule, and pathologically proven diagnoses. Up to now, there have been few cases which were diagnosed based on histological examination of living patients.^[15,30,31]^ Finally, it would be necessary to examine the risk factors for hypercoagulability, so clinicians would consider the probability of FES in advance.

## Author contributions

**Conceptualization:** So Dug Lim.

**Data curation:** Hye Seung Lee.

**Investigation:** So Dug Lim, Hye Seung Lee.

**Methodology:** So Dug Lim, Hye Seung Lee, Hong Gee Roh, Jeong-Jin Park.

**Supervision:** So Dug Lim, Hong Gee Roh.

**Validation:** So Dug Lim, Hye Seung Lee, Jeong-Jin Park.

**Writing – original draft:** Hye Seung Lee.

**Writing – review & editing:** So Dug Lim.

Hye Seung Lee orcid: 0000-0003-4157-0862.

Jeong-Jin Park orcid: 0000-0002-9456-3407.

Hong Gee Roh orcid: 0000-0003-0227-0971.

So Dug Lim orcid: 0000-0003-2036-0313.
